# Supplementary material for: Qualitative assessment of hand hygiene knowledge, attitudes and practices among healthcare workers prior to the implementation of the WHO Hand Hygiene Improvement Strategy at Faranah Regional Hospital, Guinea
Source: PLOS Glob Public Health. 2023 Feb 9;3(2):e0001581. doi: 10.1371/journal.pgph.0001581 (PMC10021640; doi:10.1371/journal.pgph.0001581)
Supplement: Attachment — Submitted filename: Response to Reviewers.docx [file pgph.0001581.s001.docx]

**PGPH-D-22-01633**

Dr Ferdinand Mukumbang

Academic Editor

PLOS Global Public Health

Manuscript reference number: PGPH-D-22-01633

Dear Editor,

We are very grateful to the Editor and the reviewers for their precious time they devoted to providing vital comments on our manuscript entitled *“Qualitative assessment of hand hygiene knowledge, attitudes and practices among healthcare workers prior to the implementation of the WHO Hand Hygiene Improvement Strategy at Faranah Regional Hospital, Guinea”*.

We have addressed these suggestions and feel that these have significantly improved our manuscript content. All the details are in our point-by-point response with each comment from the reviewers in italic font, followed by our responses.

Yours sincerely,

Moussa Douno, MD, MPH, MSc (on behalf of all co-authors).

**RESPONSE TO THE REVIEWERS**

**Journal Requirements**

*1. Please amend your detailed Financial Disclosure statement. This is published with the article. It must therefore be completed in full sentences and contain the exact wording you wish to be published.*

*a. Please clarify all sources of funding (financial or material support) for your study. List the grants (with grant number) or organizations (with url) that supported your study, including funding received from your institution. 
b. State the initials, alongside each funding source, of each author to receive each grant.
c. State what role the funders took in the study. If the funders had no role in your study, please state: “The funders had no role in study design, data collection and analysis, decision to publish, or preparation of the manuscript.”
d. If any authors received a salary from any of your funders, please state which authors and which funders.*

Response: This study was funded by the BMZ (Bundesministerium für Zusammenarbeit) as part of the GIZ (Gesellschaft für Internationale Zusammenarbeit) University and Hospital Partnerships in Africa (ESTHER) Program (Ensemble pour une Solidarité Thérapeutique Hospitalière en Réseau) (Award Number 81213469). MB received this grant. The funders had no role in study design, data collection and analysis, decision to publish or preparation of the manuscript.

*2. Since your data is not available for proprietary reasons, please explain via email why the data is not available. Please also include the contact information for the third party organization that should be contacted should other researchers want to request access to this data and please include the full citation of where the data can be found. We also request that you verify with us via email that any researcher will be able to obtain the data set in the same manner that the you have obtained it. If you feel you are unwilling or unable to adhere to this policy, please explain your reasons by return email and your exemption request will be escalated to the editor for approval. Your exemption request will be handled independently and will not hold up the peer review process, but will need to be resolved should your manuscript be accepted for publication. One of the Editorial team will be in touch if they require more information.*

Response: We are not able to make our underlying data set publicly available for ethical and data protection reasons. The data contain potentially identifying information: our data have been collected from a small group of participants, and even data that are not directly identifying in combination become identifying (e.g. sex, profession, department ward, work description). This restriction to data availability has been imposed by RKI's Data Protection Office. Data requests may be sent to the corresponding author Moussa DOUNO via [msokadouno@gmail.com](mailto:msokadouno@gmail.com).

**Reviewers’ Comments**

**REVIEWER #1:**

*Reviewer #1: This article presents a very interesting and new finding on the impact of the 2013-2016 Ebola outbreak in West Africa on hand hygiene practices, which may also provide insights for current and future pandemics. The findings are drawn from the qualitative baseline of a larger study, and as such the full data (transcripts) have not been made available. However, excerpts from the transcripts that support the author's findings are included in the results section.
In the abstract, introduce the terms in-depth interview and focus group discussion before using the acronyms.*

Response: we thank the reviewer for highlighting this missing information. We have introduced the terms “in-depth interviews” and “focus group discussions” in line 20.

*Reviewer #1: The final sentence of the abstract speaks to the results of the assessment generally, but given that this paper is on the qualitative baseline only, it would be better to highlight only those findings. I would take the last sentence out.*

Response: We thank the reviewer for highlighting the confusion caused by the word “assessment” in the sentence, which, to us, refers to the current study, not to the project’s assessment. Therefore, to avoid this confusion, we have reformulated the sentence as follows: “Our results suggest that local capacity building through training and availability of hand hygiene materials would be a sustainable approach to enhance hand hygiene culture at the hospital” (see lines 25 to 27). We have also added an additional sentence to conclude the abstract as follows: “Our study suggests that there is a need for a high and long-term commitment of authorities and healthcare workers at all levels for a sustainable hand hygiene culture” (see lines 28-29).

**Minor edits:**

*Reviewer #1: Abstract, page 1, line 19: remove the comma after "workers".*

Response: We have removed the comma after “workers” (see line 18).

*Reviewer #1: Abstract, page 1, line 22: take out "held".*

Response: We have taken out “held” in line 22.

*Reviewer #1: Abstract, page 2, line 24: take out "that is".*

Response: We have taken out “that is” in line 24.

*Reviewer #1: In the introduction, please define hand hygiene best practices and explicitly link them to health care-associated infections and patient outcomes. What are some* *examples of the ways in which other health issues are prioritized over patient safety (line 32)?*

Response: We have defined hand hygiene best practices and explicitly linked them to healthcare-associated infections and patients’ outcomes (see Lines 31-33). We have also provided some examples of the ways in which other health issues are prioritized over patient safety (see line 38-39).

*Reviewer #1: The sentence in lines 41-42 is confusing- which professional categories, duties, etc. hindered the strategy's execution, and how?*

Response: We thank the reviewer for highlighting this confusion. We have reformulated this sentence as follows:

“Certain professional categories such as doctors or nursing assistants, types and intensity of patient care, hospital wards, as well as understaffing and high workload, have been considered to be some of the factors that affect the compliance with hand hygiene” (see lines 47-51).

*Reviewer #1: The final paragraph of the introduction (lines 60-65) seems to be about the full study. It would be better to speak to the main goal of this article, which is to illuminate specific and perhaps unanticipated qualitative findings, rather than to serve as a baseline for follow-up.*

Response: We have reformulated this paragraph as follows, and think that this makes it clearer:

“The main goal of this study was to generate qualitative insights into the knowledge, attitudes and practices of healthcare workers about hand hygiene in order to inform and guide the implementation of the WHO hand hygiene improvement strategy at FRH. This qualitative data offers a baseline for follow-up research and, critically, for the planning and implementation of valid and impactful interventions at FRH. The findings are relevant not only in a post-Ebola setting for a better preparedness for epidemic diseases, but also for the prevention of healthcare associated and postoperative infections in hospital settings” (see lines 68-74).

**Minor edits:**

*Reviewer #1: Introduction, page 2, line 30: take out the hyphen in "extra-costs"*

Response: We have taken out the hyphen in “extra-costs” (see line 35).

*Reviewer #1: Introduction, page 2, lines 37-39: Simplify this sentence by saying "**The WHO strategy was first implemented on the African continent in [x year], and may have been spurred in part by the 2013-16 EVD outbreak." Specify when the strategy was implemented.*

Response: We thank the reviewer for this suggestion; we have simplified this sentence as recommended (see lines 43-45).

*Reviewer #1: Introduction, page 3, line 58: replace "whereas research has focused rather" with "and has instead focused on" (no comma after "countries").*

Response: We have done the replacement as recommended (see line 66).

*Reviewer #1: In the methods section, use darker colors for Table 1.*

Response: We have used darker colors for Table 1.

*Reviewer #1: In the data collection sub-section, clarify where the data collection activities took place (was it at the hospital?), as well as if anyone else was present other than the facilitator and participants (note-taker, third parties, etc).*

Response: We have clarified that the data collection took place at the hospital, and that no one else was present during the recording apart from the facilitator and participants (see lines 114 and 126).

*Reviewer #1: Speak to the informed consent process and how participants were recruited (face-to-face or otherwise). Was patient consent obtained for direct observations?*

Response: In the “Ethics” sub-section, we mentioned that “every participant received information about the study and signed a written consent form” (see line 159-161). In the “Study population” sub-section, we mentioned that “All participants were purposively sampled among available staff to represent various hospital units, professions and both genders” (100-101). No consent was sought from patients; we have added this to the “Ethics” sub-section as follows:

“Verbal consent was obtained from the heads of units for direct observations; no consent was sought from patients as the researcher (himself a medical doctor) attended the observed activities as a peer” (see lines 161-163).

*Reviewer #1: What was the breakdown of the 13 interviews, by participant type?*

Response: In the “Study population” sub-section, we have mentioned that “Thirteen in-depth interviews (IDIs) were conducted in the thirteen hospital wards with healthcare workers (one per ward - mainly nursing and laboratory staff) (see lines 102-103).

*Reviewer #1: How many direct observations were performed?*

Response: We have specified that 3 direct observations were performed (see line 135-136).

*Reviewer #1: On average, data collection activities took 45 minutes. Was this average different for IDIs versus FGDs?*

Response: We have reformulated as follows:

“All IDIs and FGDs were audio recorded in the presence of only the facilitator and participants with an average of 45 minutes for both” (see line 126-127).

**Minor edits:**

*Reviewer #1: Methods, page 4, line 90: replace "purposely" with "purposively".*

Response: We have replaced “purposely” with “purposively” (see line 100).

*Reviewer #1: Methods, page 4, line 93: replace "In whole" with "In total".*

Response: We have replaced “In whole” with “In total” (see line 107).

*Reviewer #1: Methods, page 4, line 105: replace "entailed" with "comprised" or "included".*

Response: We have replaced “entailed” with “comprised” (see line 118).

*Reviewer #1: Methods, page 5, line 125: replace "obtained" with "received".*

Response: We have replaced “obtained” with “received” (see line 161).

*Reviewer #1: In the results section, lines 206-208, you mention doctors noting that lack of hand hygiene during the Ebola outbreak resulted in the deaths of healthcare workers. This finding is a stark measure of risk perception and severity. Do you have supporting quotes you could add here?*

Response: We have added a supporting quote to this finding in lines 250-252 as follows:

“It is because of the negligence of hand hygiene that Ebola killed some healthcare workers elsewhere. Here at this hospital, if you’ve noticed that among all the staff, there was no Ebola cases, it is thanks to hand hygiene…”. (Nurse, IDI).

**Minor edits:**

*Reviewer #1: Results, page 6, line 127: delete "Following the main aspects of the research,".*

Response: We have deleted "Following the main aspects of the research" (see line 165).

*Reviewer #1: Results, page 7, lines 139-140: change to "ranging from washing hands with water and soap, using a 0.05% chlorine solution, to employing alcohol-based hand rubs."*

Response: We thank the reviewer for this nice rephrasing. This change has carefully been done (see lines 179-180).
*Reviewer #1: Results, page 7, lines 144-145: replace "contributing to establish" with "establishing".*

Response: We have made this replacement as recommended (see line 186).

*Reviewer #1: Results, page 7, line 152: replace "learnt a lot" with "learned more"*

Response: We have made this replacement as recommended (see line 193).

*Reviewer #1: Results, page 7, line 153: replace "response to the crisis" with "crisis response".*

Response: We have made this replacement as recommended (see line 194).

*Reviewer #1: Results, page 8, line 162: replace "Training on this form of hygiene" with "Hand hygiene training"*

Response: We have made this replacement as recommended (see line 202).

*Reviewer #1: Results, page 8, line 184-185: delete "the" in the "the protocols"*.

Response: We have deleted “the” in the “the protocols” (see line 224).

*Reviewer #1: Results, page 8, line 185-186: delete "the" in the "the necessary materials".*

Response: We have deleted "the" in the "the necessary materials" (see line 225).

*Reviewer #1: Results, page 8, line 186: add "practices" after hand hygiene*.

Response: We have added “practices” after hand hygiene (see line 226).

*Reviewer #1: Results, page 8, line 190: delete "the passage of"*

Response: We have deleted “the passage of” (see line 230).

*Reviewer #1: Results, page 9: line 193: You use "HH" for hand hygiene, but I don't see this acronym anywhere else. I would spell out hand hygiene if you are not going to use the acronym consistently.*

Response: We have spelled out “hand hygiene” as suggested (see line 233).

*Reviewer #1: Results, page 9, line 194: I would be consistent in my use of "ebola outbreak" rather than "Ebola crisis"*

Response: We thank the reviewer for this remark. We have changed “Ebola crisis” with Ebola outbreak” (see line 234):

*Reviewer #1: Results, page 9, line 195: delete "the implementation of" and "already"*

Response: We have made these deletions as recommended (see lines 235 and 236).

*Reviewer #1: Results, page 9, line 195: add "participants" after "Some"*

Response: We have added “participants” after “Some” (see line 235).

*Reviewer #1: Results, page 9, line 197: delete ").."*

Response: This has been deleted (see line 238).

*Reviewer #1: Results, page 9, line 200: add "towards hand hygiene" after "positive attitudes"*

Response: We have added “towards hand hygiene” after “positive attitudes” (see line 240-241).

*Reviewer #1: Results, page 9, lines 221-223: Why is this section italicized?*

Response: We thank the reviewer for this kind remark. We have corrected this by de-italicizing the section (see lines 267-269).

*Reviewer #1: Results, page 9, lines 223-224: Start a new paragraph with the words in bold.*

Response: We have started a new paragraph with the words in bold (see line 270).

*Reviewer #1: In the discussion section, when you talk about the "thematic model", please clarify which model you used by name, if it has one.*

Response: We are referring to the thematic model of Smiddy et al. in their article on “Systematic qualitative literature review of health care workers' compliance with hand hygiene guidelines”. The authors did not give any name to this model, that’s why we specified that it is “the thematic model deducted from a systematic qualitative review on hand hygiene by Smiddy et al.” (see lines 347-348). We have now referred to this model as “Smiddy et al.’s thematic model” throughout the discussion section.

*Reviewer #1: You don't mention the Extended Parallel Process Model (EPPM) or the Health Belief Model but I think that both could be relevant here, and would allow you to better discuss the roles of perceived severity/perceived susceptibility as well as the "cue of fear" you have referred to previously. I think incorporating these models could make the discussion section stronger.*

Response: We thank the reviewer for this valuable comment to strengthen the discussion section. We have therefore incorporated the Health Belief Model accordingly (see lines 369-377 and 411-415).

*Reviewer #1: In line 304, you mention the "use of cues" - you should connect back to this idea of the use of cues and the thematic model when you discuss the "presence of photos and posters on the walls" in the next page.*

Response: We have connected back the “use of cues” to the "presence of photos and posters on the walls" (see lines 381-384).

**Minor edits:**

*Reviewer #1: Discussion, page 12, line 307: please clarify what you mean by "acuity of care"*

Response: We have clarified this by using the full terms (Acuity of patient care) as used by Smiddy et al. in their thematic model (see line 350). This refers to the conscious or subconscious hierarchisation of care delivered to patients by HCWs as they consider some acts to have less risk than others, and thus decide not to clean their hands afterwards (see Smiddy et al. 2015).

*Reviewer #1: Discussion, page 12, line 307: replace "regimes" with "routines" or "practices"*

Response: We have replaced “regimes” with “practices” (see line 352).

*Reviewer #1: Discussion, page 12, line 318: replace "every single normal day" with "in their daily practice"*

Response: We have replaced "every single normal day" with "in their daily practice" (see line 363).

*Reviewer #1: Discussion, page 13, line 345: replace "This points" with "These findings point"*

Response: We have replaced "This points" with "These findings point" (see line 399).

*Reviewer #1: Discussion, page 13, lines 347-348: replace "is committed to monitor and supervise" with "monitors and supervises"*

Response: We have replaced "is committed to monitor and supervise" with "monitors and supervises" (see line 402).

*Reviewer #1: Discussion, page 13, line 354:* *replace "have an impact" with "has an impact"*

Response: we have replaced "have an impact" with "has an impact" (see line 408).

*Reviewer #1: Discussion, page 14, line 355: take out "HH", consider another word other than "regimes"*

Response: We have taken out “HH” and replaced “regimes” with “practices” (see line 409).

*Reviewer #1: Discussion, page 14, line 356: replace "was seen as a hindrance to the compliance" with "hindered compliance"*

Response: We have replaced "was seen as a hindrance to the compliance" with "hindered compliance" (see line 410).

*Reviewer #1: Discussion, page 14, line 356: delete "in this view"*

Response: We have deleted "in this view" (see line 416).

*Reviewer #1: Discussion, page 14, line 357: specify which materials participants were asking for*

Response: We have specified that participants were asking for “hand hygiene materials” (see line 416-417).

*Reviewer #1: Discussion, page 14, line 365: delete "Afterwards" (this is clear from "post-intervention"*

Response: We have deleted “Afterwards” (see line 424).

*Reviewer #1: Discussion, page 14, line 372: replace "This shows" with "These findings show"*

Response: We have replaced "This shows" with "These findings show" (see line 431).

**REVIEWER #2:**

*Reviewer #2: Word consistency: Health care vs. healthcare*

Response: We thank the reviewer for this remark. We have replaced “Health care” with “healthcare” throughout the text for consistency.

*Reviewer #2: Methodology:*

*Looks like it is a longitudinal follow up study. If so, mention it.*

Response: We are sorry for the confusion. This is a pre-intervention baseline assessment which informed the implementation of the PASQUALE project at Faranah Regional Hospital (see line 83-87).

*Reviewer #2: Mention no of observation, IDI and FGDs in the methodology section
If it is a purposefully sampled respondents, specify the inclusion and exclusion criteria.*

Response: We have mentioned the number of observations (see line 135-136), IDIs and FGDs (see lines 102-107). We have specified the inclusion criteria in lines 99-100.

**REVIEWER #3:**

*Reviewer #3: Lines 41-42: Unclear relationship of how certain professional categories, duties, hospital areas, and services hindered the strategy’s execution. Was it that those certain categories, duties, areas, and services were associated with lower uptake of the strategy? Any trends relevant in those association that apply to FRH?*

Response: We thank the reviewer for this observation; we have reformulated this sentence for more clarity in lines 47-51. “Certain professional categories such as doctors or nursing assistants, types and intensity of patient care, hospital wards, as well as understaffing and high workload, have been considered to be some of the factors that affect the compliance with hand hygiene”.

*Reviewer #3: Line 74: Have you triangulated and reported both the quantitative and qualitative data together in addition to the quantitative data separately?* *Demonstrating how the quantitative and qualitative data corroborate or contradict each other would strengthen the findings.*

Response: We mentioned that these findings concern uniquely the qualitative data from the pre-intervention baseline assessment, and the quantitative results were reported elsewhere (see lines 83-87). We did demonstrate how the quantitative and qualitative data corroborate in the discussion section as the quantitative findings have been already published elsewhere (see lines 423-436).

*Reviewer #3: Line 90: possible typo: should purposely be purposively?*

Response: Thank you for this remark. This typo has been corrected in line 100.

*Reviewer #3: Lines 91-97: How did you purposively select interviewees and FGD participants? Please also explain why sampling was done purposively as opposed to randomly. How was it determined whether respondents would participate in an FGD as opposed to an IDI?*

Response: We have explained how and why the sampling was done purposively in lines 100-101 and added that “The main inclusion criteria were being a healthcare worker in one of the hospital’s wards and willing to participate in the study” (see lines 99-100). We further explained how it was determined whether respondents would participate in an FGD or an IDI in lines 119-126 as follows:

“IDIs were mainly conducted with nursing and laboratory staff as they are more involved in patients’ care and sample handling, some of the acts that request more hand washing. One FGD was separately performed with each professional group (doctors, midwives, and nurses/nursing assistants/laboratory technicians) in order to avoid hierarchical influence due to differences in levels and positions between doctors and their subordinate nurses/technicians, and then encourage discussions among peers with almost the same levels and positions. Those who were individually interviewed did not participate in FGDs”.

*Reviewer #3: What was the method used for recording the observations?
Methods overall: Please include a limitations section. In this section, please also address whether participants knew they were being observed and any influence that may have had on behavior.*

Response: We have specified that the method used for recording the observations consisted of notes taking after each observation (see line 142-143). We have also added a section on the study limitations where we highlighted the influence that the study context could have had on participants’ behaviour (see lines 437-453).

*Reviewer #3: Lines 138 – 150: Consider separating out finding on knowledge of methods and practices vs knowledge that hand hygiene contribute to quality care.*

Response: Thank you for this comment, we did consider separating these, but refrained from doing it, as that would have resulted in very short sub-sections. The idea is that they mentioned the methods they know and argued that compliance with these methods enables the provision of quality care. We have reformulated it for more clarity (see lines 178-188).

*Reviewer #3: Lines 221 – 223: change in formatting with italic text was distracting.*

Response: We are sorry for this distraction; we have made the correction accordingly (see lines 267-269).

*Reviewer #3: Lines 274 – 287: How was the observation data recorded? Is there additional or more specific data on the number of incidents of HH? It is mentioned that doctors practiced HH while some nurses/nursing assistants did and some did not – is there a more specific breakdown of HH practices observed by type of employee?*

Response: We have noted that observation data were recorded by notes taking (see line 142-143). We did not do any breakdown of hand hygiene practices by type of employee as the aim of the observation was not to see who does or does not practice hand hygiene. Rather, it was to see whether healthcare workers take initiative to practice hand hygiene as they were saying in interviews/discussions (see lines 127-143).

*Reviewer #3: Results overall: I think the results would be strengthened by including the quantitative data from the baseline to better understand how health workers knowledge, attitudes, and beliefs reinforces or contradicts reported and observed behaviors.*

Response: We are sorry for the confusion, but the quantitative data have been published elsewhere; these findings concern only the qualitative baseline assessment (see lines 83-87). Therefore, we demonstrated in the discussion section how the findings of this qualitative study correlate to those of the quantitative ones already published (see lines 423-436).

*Reviewer #3: Lines 301 – 307: Did this theoretical model inform the development of study design and data collection tools? If so, specify in the methodology section.*

Response: We are sorry for this confusion. No, this theoretical model did not inform the development of the study design and data collection tools, that’s why it is not referred to in the methodology section.

*Reviewer #3: Line 365: Describe in more detail in what ways PASQUALE responded directly to the findings of this study and the quantitative baseline.*

Response: We think that in the same paragraph, we mentioned the local production of alcohol-based hand rub, training sessions and supervision that the project implemented (see lines 427-436). We have also referred to “S. A. Müller *et al.*, Implementation of the WHO hand hygiene strategy in Faranah regional hospital, Guinea” for more details on PASQUALE project implementation.

*Reviewer #3: Overall: please include copies of the interview and FGD tools as an annex.*

Response: We have taken note of this request, we will check whether the journal accepts the interview guides as an annex.

**REVIEWER #4:**

*Reviewer #4: This paper reports on healthcare providers’ hand hygiene knowledge, attitudes, and practices prior to implementation of the WHO strategy at Faranah Regional Hospital. The qualitative exploration of knowledge, attitudes, and practices holds value for informing efforts to prevent healthcare-associated infections. However, the manuscript includes limited methodological detail, making it challenging to determine the methodological rigor of the study. Additionally, the findings are not clearly delineated and would benefit from significant reorganization.
Below are specific recommendations that could help strengthen the paper:
Reviewer #4: More detail on the observation procedures, including how many people were observed, with what frequency, and the total duration of the observation, would be useful. Additionally, how were the observation data documented and analyzed?*

Response: We thank the reviewer for his valuable comments. We have integrated more details on the observation procedures (see lines 127-143), and stated how the observation data were documented and analyzed (see lines 153-154).

*Reviewer #4: The authors reference thematic analysis, however, there is no description of the analytic process beyond the development of a codebook. Please detail how transcripts were coded (e.g. in duplicate?, how were discrepancies resolved?), how coded data were then analyzed and organized into themes, and any rigor steps taken to strengthen the analysis.*

Response: We have reviewed the section on data analysis and provided more details on the analytic process as follows (see lines 144-157):

**“Data analysis**

Sociodemographic data of the respondents were descriptively summarized in means (see table 2). By assigning a code to each of the thirteen IDIs and three FGDs, participants were de-identified. Audio recordings were transcribed verbatim, and all transcripts were cross-checked for consistency to enable thematic analysis. The initial analytical coding frame was developed following the main points of the topic guide and was performed using Microsoft Word for Windows 10 (deductive coding). The recurrent themes that emerged from the data were used to refine the initial codebook (inductive coding). Based on that, a thematic content analysis was then conducted through line-by-line reading of all the transcripts, and the results were informed by concepts grounded in the data. Notes from the observations were descriptively elaborated to form a sub-section of the study results. Fieldwork, transcription and analysis were carried out in French; verbatim quotations were translated into English by MD for the purpose of this paper”.

*Reviewer #4: The authors mention data saturation, but do not describe how they assessed saturation or whether the sample size was driven by, or independent of, saturation. Please describe how and when saturation was addressed, e.g. were data collection and analysis conducted concurrently or was saturation assessed after the fact?, and what approaches as used to determine the point at which saturation was achieved?*

Response: We thank the reviewer for highlighting this confusion. We have taken out the sentence on data saturation as it was not in the appropriate section. Data saturation should have been mentioned in the section on data collection, but we did not refer to it because we did not aim at data saturation in this assessment. Rather, we purposively selected an equal number of healthcare workers in each hospital ward (one per ward) for IDIs, and performed one FGD with each professional category (doctors, midwives, and nurses/nursing assistants/lab technicians) (see lines 102-107).

*Reviewer #4: The authors state that the results are presented in three categories: knowledge of hand hygiene, attitudes toward hand hygiene, and hand hygiene practices. However, the results are presented in four sections: (1) knowledge, (2) attitudes, and (3) barriers and facilitators to hand hygiene, which also includes suggested improvements; and (4) hand hygiene practices. Clearly outlining the presentation of findings will help orient the reader.*

Response: We thank the reviewer for his vigilance on this. We have corrected by clearly delineating the themes and sub-themes. “Facilitators and barriers to hand hygiene practices” and “observed practices” are sub-themes of “Practices” which is the main theme, the third category (see lines 254 and 320).

*Reviewer #4: The knowledge, attitudes, and barriers and facilitators sections lack clear differentiation. For example, the findings related to ‘learning from the Ebola epidemic,’ and ‘the influence of the Ebola epidemic,’ are not meaningfully differentiated, and the section on the influence of the ebola epidemic, in particular, weaves between how healthcare workers became knowledgeable about hand hygiene, and how their attitudes toward hand hygiene were affected by the 2013-2016 Ebola outbreak. Additionally, the section on facilitator and barriers to hand hygiene practices weaves between priorities and values that shape the attitudes of healthcare works (e.g. self protection, avoiding resurgence) and tangible things like the presence of a hospital hygiene committee or the absence of soap, running water, etc. The manuscript would benefit from a restructuring of the presentation of findings to address areas of overlap and improve clarity of the major themes and sub-themes.*

Response: We are sorry for this confusion in the results section, we think that with the clear delineation of the themes and sub-themes, together with the consideration of previous comments from the other reviewers on this section, this overlap has been addressed to our best. Given that the three other reviewers agreed to the outlined structure, we would refrain from changing it considerably, but we have addressed previous comments by integrating corrections which convey the concepts from the data.

*Reviewer #4: Did the authors consider differences in knowledge and attitudes based on profession? It would be interesting to explore and address any differences* *between medical doctors, midwives, nurses, and nursing and laboratory assistants. Additionally, as written, the illustrative quotations disproportionally represent doctors’ views, while midwives, nursing and laboratory assistants’ voices are not well represented. The manuscript would be strengthened by the addition of more representative quotes, either in the text or in a supplementary table.*

Response: We agree that differences based on professions would be interesting. However, the present baseline assessment only aims at having an overview of healthcare workers’ knowledge, attitudes and practices in general. We have therefore specified that we did not consider differences in knowledge and attitudes based on profession (see lines 167-169). But doctors gave more elaborative assertions (probably due to their higher level) than other participants, that’s why more illustrative quotes came from them.

*Reviewer #4: The observed practices section would benefit from some quantification. For example, how frequently was hand washing practiced versus not practiced by nurses and nursing assistants? As currently written, this section is not enhancing the manuscript.*

Response: We have addressed this by providing more details and specifying the number of observations performed (see lines 127-143 and line 331).
